# Essential Role of Thioredoxin 2 in Mitigating Oxidative Stress in Retinal Epithelial Cells

**DOI:** 10.1155/2013/185825

**Published:** 2013-11-10

**Authors:** Eriko Sugano, Namie Murayama, Maki Takahashi, Kitako Tabata, Makoto Tamai, Hiroshi Tomita

**Affiliations:** ^1^Department of Chemistry and Bioengineering, Faculty of Engineering, Graduate School of Engineering, Iwate University, 4-3-5 Ueda, Morioka, Iwate 020-8551, Japan; ^2^School of Medicine, Tohoku University, 2-1 Seiryou-machi, Sendai 980-8574, Japan; ^3^Graduate School of Medicine, Tohoku University, 2-1 Seiryou-machi, Sendai 980-8574, Japan; ^4^Clinical Research, Innovation and Education Center, Tohoku University Hospital, 2-1 Seiryou-machi, Sendai 980-8574, Japan; ^5^Laboratory of Visual Neuroscience, Department of Chemistry and Bioengineering, Faculty of Engineering, Graduate School of Engineering, Iwate University, 4-3-5 Ueda, Morioka, Iwate 020-8551, Japan

## Abstract

The retina is constantly subjected to oxidative stress, which is countered by potent antioxidative systems present in retinal pigment epithelial (RPE) cells. Disruption of these systems leads to the development of age-related macular degeneration. Thioredoxin 2 (Trx2) is a potent antioxidant, which acts directly on mitochondria. In the present study, oxidative stress was induced in the human RPE cell line (ARPE-19) using 4-hydroxynonenal (4-HNE) or C2-ceramide. The protective effect of Trx2 against oxidative stress was investigated by assessing cell viability, the kinetics of cell death, mitochondrial metabolic activity, and expression of heat shock proteins (Hsps) in Trx2-overexpressing cell lines generated by transfecting ARPE cells with an adeno-associated virus vector encoding Trx2. We show that overexpression of Trx2 reduced cell death induced by both agents when they were present in low concentrations. Moreover, early after the induction of oxidative stress Trx2 played a key role in the maintenance of the cell viability through upregulation of mitochondrial metabolic activity and inhibition of Hsp70 expression.

## 1. Introduction

Retinal epithelial (RPE) cells perform multiple functions to maintain retinal homeostasis, including preserving the blood-retinal barrier, nourishing retinal cells by secreting growth factors [1, 2], phagocytosis of shed photoreceptor outer segments [3, 4], and maintenance of the visual cycle by resynthesizing 11-cis retinal [5]. RPE cells located anterior to photoreceptors endure significant oxidative stress because they consume high levels of oxygen and polyunsaturated lipids and are subjected to long-term exposure to light [6]. To protect against oxidative stress, RPE cells employ antioxidant systems, involving glutathione (GSH) S-transferases (GST), heme oxygenase-1 (HO-1), superoxide dismutase (SOD), peroxiredoxin (PRDX1) [7], and thioredoxin (Trx) [8]. Under physiological conditions, the intracellular redox potential is maintained by the synthesis of high concentrations of GSH. However, exposure to high levels of reactive oxygen species (ROS) or free-radical-generating molecules can alter the redox balance. Sulfhydryl groups are critical for the response to oxidative stress, and thioredoxin maintains cellular redox potential [3, 9, 10].

Trx, originally identified in *Escherichia coli* as a hydrogen donor for ribonucleotide reductase [11], contains two conserved cysteine residues within its active site [12, 13] and scavenges intracellular ROS. Oxidized Trx is reduced by Trx reductase in the presence of NADPH. Thus, Trx maintains the function of metabolic enzymes whose catalytic activity depends on the presence of disulfide bonds. These enzymes repair proteins damaged by oxidation, reduce dehydroascorbate, metabolize sulfur, ensure proper polypeptide chain folding, and regulate protein function.

Thioredoxins are present in all living cells. Two classical Trx isoforms, including Trx1 in the cytosol/nucleus and Trx2 in mitochondria, are both essential, and inactivating mutations in Trx genes are embryonically lethal [14]. We demonstrated the protective effect of Trx2 against oxidative stress caused by 4-hydroxy nonenal (4-HNE) that involves upregulation of I*κ*B activity through its phosphorylation and translocation of NF-*κ*B to the nucleus [8]. Further, Trx2 prevents mitochondrial membrane depolarization. These findings led us to conclude that Trx2 is localized to mitochondria, which are highly vulnerable to oxidative damage. However, Trx2 had no effect on cell death caused by high concentrations of 4-HNE. Specifically, 50 *μ*M 4-HNE induced 78% cell death and 41% at 25 *μ*M, and Trx2 only protected against relatively low concentrations (≤25 *μ*M) of 4-HNE. Therefore, there may be a fundamental difference in how oxidative stress affects cells in the presence of 50 *μ*M or 25 *μ*M 4-HNE.

In the present study, we investigated whether Trx2 protects against oxidative stress caused by 4-HNE or C2-ceramide in the human retinal pigmented epithelial cell line (ARPE-19). We also studied whether exponential cell death caused by 4-HNE as a function of its concentration is common in oxidative stress and how Trx2 affects this process.

## 2. Materials and Methods

### 2.1. Cell Culture

The human ARPE-19 (ARPE) cell line was provided by L. Hjelmeland (Department of Ophthalmology, Section of Molecular) and was maintained in Dulbecco's modified Eagle's medium (DMEM) supplemented with 10% fetal bovine serum (FBS) and antibiotics, all from Life Technologies (Tokyo, Japan) as described previously [3, 15]. The medium was changed every three days, and cultured cells were passaged after treatment with a solution containing 0.125% trypsin and 0.01% EDTA. The AAV-293 cell line was obtained from Agilent Technologies (Tokyo, Japan), and HT1080 cells were supplied by the Cell Resource Center of Tohoku University (Sendai, Japan) and were maintained in DMEM supplemented with 10% FBS and passaged with 0.05% trypsin-0.53 mM EDTA or 0.125% trypsin-0.01% EDTA, respectively. Cultured cells were incubated in a humidified incubator in an atmosphere containing 5% CO_2_.

### 2.2. Preparation of an Adeno-Associated Virus (AAV) Vector Carrying Trx2 Constructs

An adeno-associated virus (AAV) vector carrying Trx1 or Trx2 was constructed as previously described [8]. A control vector was generated by inserting only pmCherry coding sequences. The pAAV-RC and pHelper plasmids were obtained from the AAV Helper-Free System (Agilent Technologies, Tokyo, Japan).

### 2.3. Production of Recombinant AAV Vectors and Infection

The AAV vectors were produced according to a published method [16]. To determine the virus titer, the level of AAV2-specific capsid proteins was measured using an enzyme-linked immunosorbent assay (Progen Biotechnik, Heidelberg, Germany), and virus titer was defined as number of capsids per mL [17]. ARPE cells were infected according to the manufacturer's instructions (Agilent Technologies). Successful transgenic expression of Trx1 or Trx2 was confirmed by assessing the expression of pmCherry fluorescence by microscope (Axiovert 40; Carl Zeiss, Oberkochen, Germany) and confocal microscope (LSM700; Carl Zeiss). The localization of the Trx2 in the cells was also further investigated using MitoTracker dye (Life Technologies) as a mitochondrial marker.

### 2.4. Cell Viability Assay

ARPE cells were plated on a 96-well plate. One day after plating, the medium was exchanged for a medium containing various concentrations of 4-HNE (EMD Millipore, Billerica, MA, USA) or C2-ceramide (Enzo Life Sciences, Tokyo, Japan) to induce oxidative stress in the absence of FBS. After incubating the cells for 3, 6, and 24 hours, their viability was assessed by measuring the reduction of 3-(4,5-dimethlthiazol-2-yl)-5-(3-carboxymethoxyphenyl)-2-(4-sulfophenyl)-2H-tetrazolium, inner salt (MTS) to an insoluble formazan (MTS assay, CellTiter 96 AQueous Non-Radioactive Cell Proliferation Assay, Promega, Madison, WI, USA). To study the effect of Trx1 or Trx2 on stress, ARPE cells were infected with AAV-Trx1, AAV-Trx2, or AAV-pmCherry. After three days, the expression of pmCherry fluorescence was confirmed using fluorescence microscope, and the cells were added to the wells of a 96-well plate. One day after plating, oxidative reagents were added, and their effects on cell viability were determined using the MTS assay described above.

### 2.5. Analysis of Cell Proliferation

Uninfected ARPE cells and cells infected with AAV-Trx1 or -Trx2 were plated on a 24-well culture plate at 1 × 10^4^ cells/well, and the number of cells was counted at 6, 24, and 48 hours after seeding to the plate using a TC20 Cell Counter (Bio-Rad, Hercules, CA, USA).

### 2.6. Immunoblot Analysis

Cells were exposed to 25 *μ*M of 4-HNE or medium for 3 hours in the absence of FBS and were then lysed with RIPA buffer (Thermo Fisher Scientific, Suwanee, GA, USA) and kept on ice for 5 min. The lysates were centrifuged, and the supernatant was used for western blot analysis according to the manufacturer's protocol. Twenty-five micrograms of the lysate was subjected to 4–15% SDS-PAGE (Mini-PROTEAN system, Bio-Rad), and the proteins were transferred to a polyvinylidene difluoride membrane (Bio-Rad). The blots were probed with antibodies against Hsp70 and Hsp90 (Cell Signaling Technology, Boston, MA, USA) and *β*-actin (Santa Cruz Biotechnology, Inc., Santa Cruz, CA, USA). For reblotting with primary antibodies, Re-Blot Plus (Millipore, Darmstadt, Germany) was used to strip the proteins from the membranes. Protein bands were detected using a Flour-S MAX Chemiluminescence Imager (Bio-Rad). Densitometric analysis of these proteins was performed using Quantity One software (Bio-Rad).

### 2.7. Statistical Analysis

Statistical analysis was performed using GraphPad Prism (GraphPad Software, San Diego, CA, USA). The unpaired *t*-test or Bonferroni's multiple comparison test was used to assess differences in cell viability or mitochondrial membrane potential, respectively. Statistical significance was defined as *P* < 0.05.

## 3. Results and Discussion

### 3.1. Results

#### 3.1.1. Kinetics of Cell Death under Oxidative Stress

**Figure 1:**
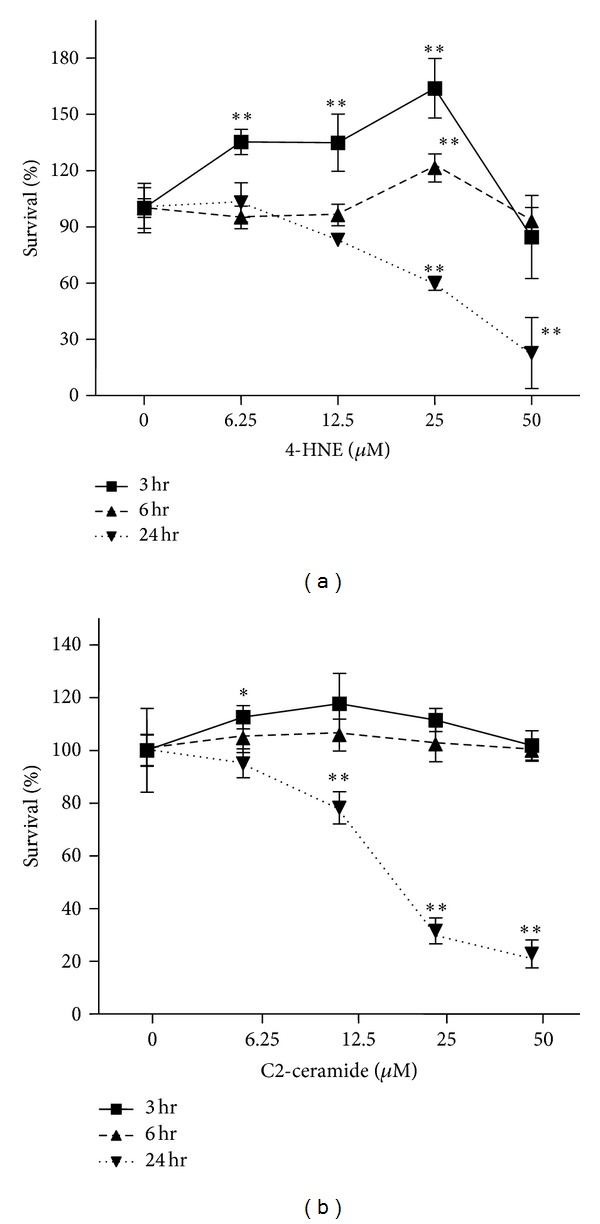
Kinetics of cell death under oxidative stress. Uninfected ARPE cells were exposed to 4-HNE or C2-ceramide. Cell survival was measured by MTS assay after 3, 6, or 24 hours of exposure. Cells not exposed to the stressors served as controls. Data represent the mean ± standard deviation (SD) (*n* = 5, *P < 0.05; **P < 0.01; unpaired *t*-test).

After three, six, and 24 hours of exposure to 4-HNE or C2-ceramide, using the MTS assay, the survival of untreated cells was compared with that of treated cells. Oxidative stress increased cell survival three hours after exposure. After six hours, there was a little protective effect against 4-HNE- or C2-ceramide-induced stress. After 24 hours of exposure, both agents caused significant cell death in a dose-dependent manner (Figure 1).

#### 3.1.2. Expression of Trx1 or Trx2 in ARPE Cells

**Figure 2:**
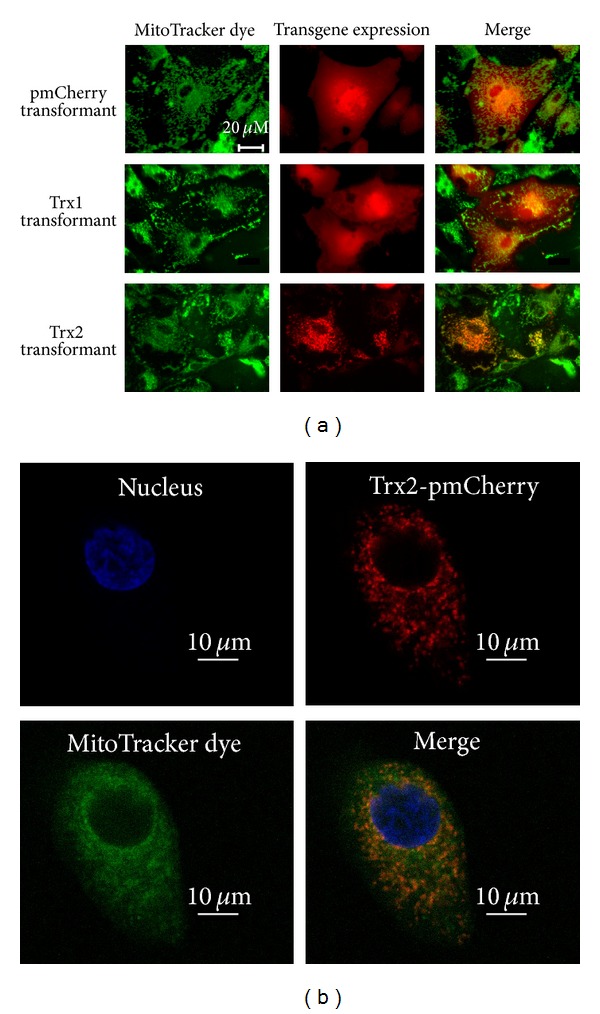
Expression profile of Trx1 and Trx2 in ARPE cells. ARPE cells were infected with AAV-Trx1 or AAV-Trx2, and Trx-overexpressing cells were established (ARPE-Trx1, ARPE-Trx2). The expression of Trx1 and Trx2 is indicated by the fluorescence of the reporter protein pmCherry, which was fused to Trx. Localization of Trx2 to mitochondria was examined by the fluorescence of a mitochondrial marker dye. ARPE cells infected with AAV-pmCherry served as a control. Fluorescence was observed using microscope (a) and confocal microscope (b).

The expression of the transgenes was observed using a fluorescence microscope. Expression of pmCherry and Trx1-pmCherry was observed in the cytoplasm, while that of Trx2-pmCherry mainly colocalized with a mitochondrial marker, MitoTracker dye (Figures 2(a) and 2(b)), which is consistent with the localization of endogenous Trx2 in ARPE cells.

#### 3.1.3. Proliferation of Cells Infected with Trx Expression Vectors

**Figure 3:**
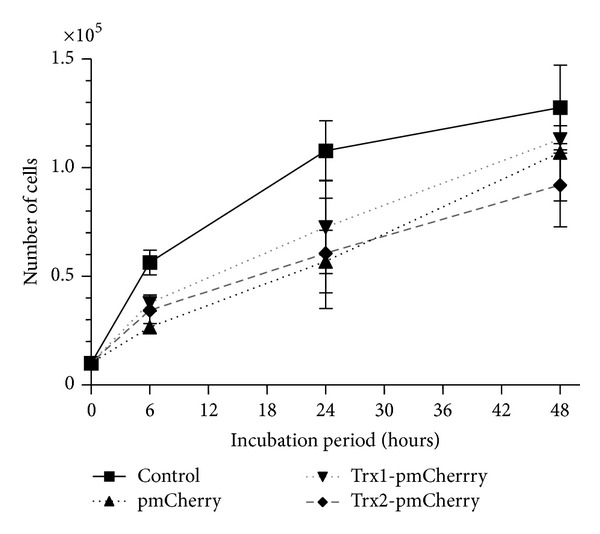
Proliferation analysis. Uninfected ARPE cells and cells infected with AAV-Trx1 or AAV-Trx2 were plated on a 24-well culture plate at 1 × 10^4^ cells/well. The number of the cells was counted at 6, 24, and 48 hours after seeding using a TC20 Cell Counter.

Cell proliferation was determined by counting the cells. The transgene reduced cell proliferation; however, there was no significant difference between infected and uninfected cells (Figure 3).

#### 3.1.4. Protective Effect of Trx2 on Cell Survival

**Figure 4:**
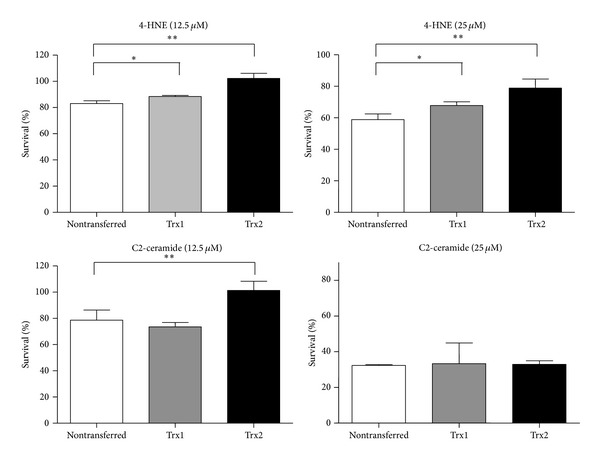
Protective effect of Trx2 against oxidative stress. ARPE-Trx1, ARPE-Trx2, ARPE-pmCherry, and uninfected ARPE cells were exposed to 4-HNE or C2-ceramide. After 24 hours of incubation, cell viability was assessed using the MTS assay. Untreated ARPE cells were also analyzed and their number was defined as 100% survival. Data represent the mean ± SD (*n* = 5, *P < 0.05; **P < 0.01; unpaired *t*-test).

We used 4-HNE or C2-ceramide to induce oxidative stress in ARPE cells expressing transgenic Trx1 or Trx2. Trx2 protected cells exposed to 12.5 *μ*M and 25 *μ*M of 4-HNE as well as to 12.5 *μ*M of C2-ceramide but not those exposed to 25 *μ*M C2-ceramide (Figure 4). The protective effect by Trx1 was less than that of Trx2.

#### 3.1.5. Immunoblot Analysis

**Figure 5:**
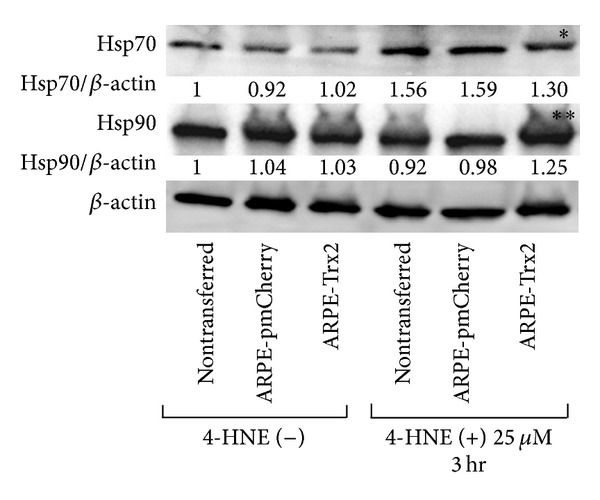
Western blot analysis of heat shock proteins expressed by cells under oxidative stress. Cells were exposed to 4-HNE for 3 hours. Untreated cells served as controls. The same blots were reprobed with an anti-*β*-actin antibody to serve as loading controls. Hsp70 (∗) or Hsp 90 (∗∗) expression in ARPE-Trx2 cells was compared with that of untransfected or ARPE-pmCherry cells that were exposed to 4-HNE for 3 hours. Densitometric analysis of these proteins was performed using Quantity One software. The value relative to *β*-actin of untreated cells is defined as 1.

Hsp90 expression was increased in cells expressing Trx2 three hours after exposure to 4-HNE (Figure 5). Hsp70 expression increased three hours after exposure to 4-HNE in all cells; however, its expression in ARPE-Trx2 cells was lower compared with uninfected and ARPE-pmCherry cells.

### 3.2. Discussion

We show here the relation between Trx-mediated protection against oxidative stress and Hsp expression. We used 4-HNE and C2-ceramide to induce oxidative stress. Trx2 protected cells from oxidative stress caused by low but high concentrations of stressors, indicating that different pathways of cell death were involved. The oxidative stressor 4-HNE is a diffusible aldehyde product of membrane-lipid peroxidation (LPO), which produces relatively stable and toxic electrophiles. Moreover, 4-HNE may act as a key mediator of oxidative-stress-induced cell death [18]. Elevated levels of 4-HNE were detected in patients with oxidative-stress-related degenerative diseases [19]. In the retina, ROS-induced LPO of polyunsaturated fatty acids in RPE cells produces higher amounts of LPO products, including 4-HNE. Kapphahn et al. reported that 4-HNE is a major oxidant in the retina [20, 21].

Ceramide is an endogenous mediator of apoptosis, and when its intracellular concentration is increased under oxidative stress [22] cell proliferation is inhibited and cell death is induced. Ceramide is converted by ceramidases to sphingosine, the levels of which increase in the early steps of apoptosis [23]. In the retina, photoreceptors are susceptible to ceramide [24], and during the phagocytosis of photoreceptors ceramide attacks RPE cells. In the present study, we used *N*-acetylsphingosine (C2-ceramide), a synthetic cell-permeable ceramide analog.

Here we show that oxidative stress induced by both stressors caused cell death in a concentration-dependent manner (Figure 1). Our previous study demonstrated that Trx2, a mitochondrial redox protein, rescued cells from 4-HNE-induced oxidative stress and showed that it plays a key role in mitochondrial function [8]. Gudz et al. reported that C2-ceramide caused mitochondrial dysfunction through direct reduction of the mitochondrial respiratory chain complex III [25]. Thus, we hypothesized that Trx2 exerts a protective effect against C2-ceramide-induced oxidative stress. This hypothesis is supported by our present data showing that Trx2 protected ARPE cells from the effects of exposure to 12.5 *μ*M C2-ceramide, indicating that the Trx2 protects mitochondrial function (Figure 4). Consistent with our data published previously [8], Trx1 expressed in the cytoplasm was less protective against C2-ceramide-induced oxidative stress than Trx2.

In contrast, Trx2 did not protect cells from exposure to 25 *μ*M C2-ceramide. Figure 1 shows that oxidative stress induced by a high concentration of C2-ceramide caused rapid cell death. The differences in the cellular response at the threshold concentrations may involve either apoptosis [26, 27] or necrotic cell death [28]. In the present study, we attempted to determine whether Trx2 protected against oxidative stress induced by a low concentration of 4-HNE.

To study the effects of oxidative stress on ARPE cells, we measured the activity of mitochondrial dehydrogenases using the MTS assay. These results show that low concentrations of oxidative stressors upregulated mitochondrial function early after exposure (three to six hours) but not at 24 hours (Figure 1). Therefore, we assume that 3 hours of exposure caused protection and 24 hours of exposure led to excess stress. Thus, if Trx2 functionally affected cell survival only at a low concentration, 3 hours may be the key time point to rescue the cells. Thus, we focused on endoplasmic reticulum (ER) stress because of its function as a double-edged sword to combat environmental insults and intrinsic stress. Salminen et al. suggested that ER stress plays a potent role in age-related macular degeneration (AMD) [29].

In ER stress, cells activate a self-protective system termed the unfolded protein response, which includes elevation of molecular chaperone expression and ER-associated protein degradation. In contrast, excessive stress activates apoptosis. We hypothesized that ER stress activates mitochondrial metabolic activity early after exposure to 4-HNE, and prolonged exposure (24 hours) causes ER-mediated cell death. To study ER stress induced by a low concentration of 4-HNE (25 *μ*M), expression of ATF4 and phosphorylated-JNK (p-JNK) was analyzed using western blotting. However, 4-HNE did not induce ATF4 expression and did not increase p-JNK expression (data not shown).

To study ER stress caused by misfolding, we analyzed Hsp expression. Hsps prevent the accumulation of cytotoxic protein aggregates and assist in the refolding of misfolded proteins. Hsp90 is one of the most abundant proteins in eukaryotic cells, and its expression can increase severalfold after stress [30, 31]. Hsp70 is involved in cell fate control [32]. Both Hsps regulate the activation of the HSF1 transcription factor, which is linked to the transcriptional stress response [33, 34]. Kaarniranta et al. reported that 4-HNE induces the expression of Hsps [35]. Therefore, we determined whether these Hsps are involved in the protective effects of Trx2 and found that 4-HNE increased Hsp90 expression but inhibited the increase of Hsp70 expression. Kaarniranta et al. reported that inhibition of Hsp90 led to an increase of Hsp70 expression and cell damage [35]. Thus, inhibition of Hsp70 by Trx2 might protect against oxidative stress, and its protective effect was limited to the stress caused by a low concentration of 4-HNE. However, our experiments were performed using an overexpression model, and several nonspecific reactions might have occurred. Further experiments will be required to reveal how endogenous Trx2 functions to counteract oxidative stress.

## 4. Conclusion

Oxidative stress is a trigger for AMD. In the present study, two inducers of oxidative stress, 4-HNE and C2-ceramide, were added to ARPE cells, and the protective effect of Trx2 expression was investigated. Both stressors caused cell death in a concentration-dependent manner and induced rapid cell death at a high concentration. These results suggest that there are concentration-dependent differences in the types of cell death. Trx2 only protected ARPE cells from low concentrations that upregulated mitochondrial activity early after exposure. Trx2 protected cells by inhibiting the increase in Hsp70 expression induced by oxidative stress.
